# Case report: Flexor carpi ulnaris tendinopathy in a lure-coursing dog treated with three platelet-rich plasma and platelet lysate injections

**DOI:** 10.3389/fvets.2023.1003993

**Published:** 2023-01-19

**Authors:** Alessio Franini, Maria Grazia Entani, Elisa Colosio, Luca Melotti, Marco Patruno

**Affiliations:** ^1^Sporty Dog Veterinary Clinic, Brescia, Italy; ^2^Department of Comparative Biomedicine and Food Science, University of Padua, Padua, Italy

**Keywords:** platelet-rich plasma (PRP), platelet lysate (PL), dog, tissue regeneration, regenerative medicine, canine orthopedics

## Abstract

In the present case report a 7-year-old male Whippet competing in lure-coursing presented with third-degree recurrent lameness of the right forelimb, pain on palpation of the caudal aspect of the carpus and swelling of the forearm proximally to the accessory carpal bone. Clinical, radiographic, and ultrasonographic evaluation diagnosed a flexor carpi ulnaris (FCU) chronic tendinopathy unresponsive to previously attempted conservative treatments such as oral non-steroidal anti-inflammatory drugs (NSAIDs) administration along with padded palmar splint application and rest. The dog was subjected to one injection of autologous platelet-rich plasma (PRP) obtained using a double centrifugation tube method, followed by two platelet lysate (PL) injections. Treatment was administered at three-week intervals. The healing process was assessed through clinical and ultrasonographic imaging (US) on the day of the first injection (T0), and at week three (T1), six (T2), twelve (T3), fifty-two (T4), and one-hundred-and-four (T5). Fiber alignment score (FAS) and echogenicity score (ES) were developed by modifying a previously published US assessment scale. At T1, ES, and FAS improvement was detected, and at T2, further improvements in ES and FAS were observed. Ultrasonographic results were clinically consistent with the improvement in lameness: lameness grade 3/4 was detected at T0 and grade 2/4 at T1. A lameness grade of 1/4 was detected at T2, and grade 0/4 was observed at T3, T4, and T5. Moreover, at T5, the dog returned to competition, and no history of re-injury was reported. Our results suggest that the treatment of FCU tendinopathy in lure-coursing dogs with a combination of consecutive injections of autologous PRP and PL could be feasible. Additionally, no adverse reactions were observed.

## 1. Introduction

The flexor carpi ulnaris (FCU) muscle in dogs acts as a flexor and an abductor of the forepaw. It also has an antigravitational function. It consists of two bellies: the ulnar and humeral heads. Both end distally on the accessory carpal bone with two tendons. The ulnar head extends distally with a flat tendon laying lateral and palmar to the superficial digital flexor muscle, covering the humeral head. Progressing distally, it ends at the apex of the accessory carpal bone, which is separated from the humeral head tendon. The humeral head tendon is short, strong, and inserts into the apex of the accessory carpal bone (1).

Although only occasionally reported, lesions of the actual tendon itself have been described in Coursing dogs as typical sport-related injuries, and lesions of the insertion of the FCU tendon have been described in veterinary sports medicine as a consequence of strain followed by inadequate rest (2). A single case report on a Weimaraner was described in Kuan et al. (3) assuming that insertional tears of the FCU tendon have to be included in the differential diagnosis of thoracic limb lameness and peri-carpal swelling.

In human sports medicine, wrist and hand injuries reportedly affect a large part of the sporting population, and FCU tendinopathy is described as an overuse injury involving sports with a high potential of wrist and hand trauma such as tennis, handball, netball, or basketball (4).

The diagnosis of hand injuries in human medicine is considered challenging, and the importance of ultrasonographic evaluation of musculoskeletal diseases is increasing with applications in wrist injuries (5, 6). In veterinary medicine, ultrasound (US) is considered an affordable and available imaging technique that allows dynamic evaluation of anatomical structures, leading to subsequent examinations, thus providing an evaluation of the healing process of injured tendons and muscles. Although not considering the palmar face, carpal ultrasound has recently been described as a feasible imaging method for the assessment of tendinous structures of this highly complex joint (7).

The treatment of injuries caused by the insertion of the FCU tendon in humans has been reported to be conservative or surgical. The first consists of non-steroidal anti-inflammatory medications, splints, therapeutic strengthening, stretching programs, and steroidal injections. In contrast, surgical treatment is applied to patients who fail conservative management and has been reported as excision of the pathological tissue of the tendon (8).

In veterinary medicine, surgical treatment of injuries to the FCU tendon at its insertion has been reported in cases of avulsion from the accessory carpal bone providing suture of the damaged portion, splint in flexion of the carpus for 2 or 3 weeks, and restriction of activity for at least 6 weeks (2). Conservative management has been described as providing a padded splint for 3 weeks, oral administration of non-steroidal anti-inflammatory medications, and topical injections of steroids (3).

Although not described in the specific circumstances of FCU tendinopathy, the management of tendon lesions with regenerative medicine treatments appears to be a promising approach in human medicine. Among the regenerative strategies, the application of platelet-derived products, such as platelet-rich plasma (PRP) and platelet lysate (PL), has gained interest because of the concentrated content of bioactive factors, which is correlated to tissue healing properties. These factors include cytokines and growth factors, such as transforming growth factor-β (TGF-β), insulin-like growth factor 1 (IGF-1), platelet-derived growth factor (PDGF), vascular endothelial growth factor (VEGF), and basic fibroblast growth factor (bFGF), that by acting individually or in synergy can promote cell proliferation, cell migration, and recruitment along with angiogenesis, hence initiating a healing response within the damaged tissue (9–13). These products can be obtained with minimally invasive procedures and can be cryopreserved for multiple applications in a long-term perspective (9, 14). In Veterinary Medicine, the regenerative strategy to treat tendon injuries started to be studied in the nineties in the field of equine sports medicine, progressing during the years and gaining visibility in recent times also in the field of canine sports medicine, being described for the treatment of different orthopedic conditions in dogs (15, 16).

The canine flexor tendon has been studied as an animal model for flexor tendon healing and repair after different types of injuries for treatment in humans (17). Thus, the application of regenerative approaches for FCU tendinopathy in dogs may be a useful tool for providing preclinical data for future applications in human athletes.

In this study, a single case of FCU tendinopathy in a lure-coursing dog that was not responsive to conservative treatments and treated with multiple injections of autologous PRP and PL is described along with an ultrasonographic follow-up of the healing process 2 years after treatment.

## 2. Case description

### 2.1. Clinical history

A 7-year-old male Whippet competing in lure-coursing was referred to our hospital with a history of third-degree recurrent lameness to the right forearm. Symptoms were noticed 3 months before the clinical evaluation, and analog episodes were reported during the previous 3 years. Lameness was evident during walking, and it was worsening during sports activity as reported by the owner. No history of trauma was reported.

Conservative treatment with rest was previously suggested, and only a short leash walk was allowed. A padded palmar splint was administered and 4 mg/kg Carprofen was orally administered once a day for 2 weeks (18). No response to conservative treatment was achieved, and no improvements were observed at the time of the splint removal.

On clinical examination, the dog was found to be in good general health. Orthopedic examination showed third-degree right forelimb lameness, based on a previously reported orthopedic lameness evaluation system (19) (Table 1). Pain upon palpation of the caudal aspect of the right carpus and hyperextension of the same joint were detected. Swelling proximal to the accessory right carpal bone has also been previously reported. The range of motion of the carpal joint was bilaterally normal with mild pain reaction to right carpal hyperextension. The varus/valgus stress tests were non-pathologic in both thoracic limbs, with stable carpal joints.

**Table 1:** Lameness evaluation system (19).

**Grade**	**Degree of lameness**
1	No lameness
2	Intermittent weight-bearing lameness
3	Permanent weight-bearing lameness
4	Non weight-bearing lameness

### 2.2. Diagnostic imaging: Radiographic examination

**Figure 1:**
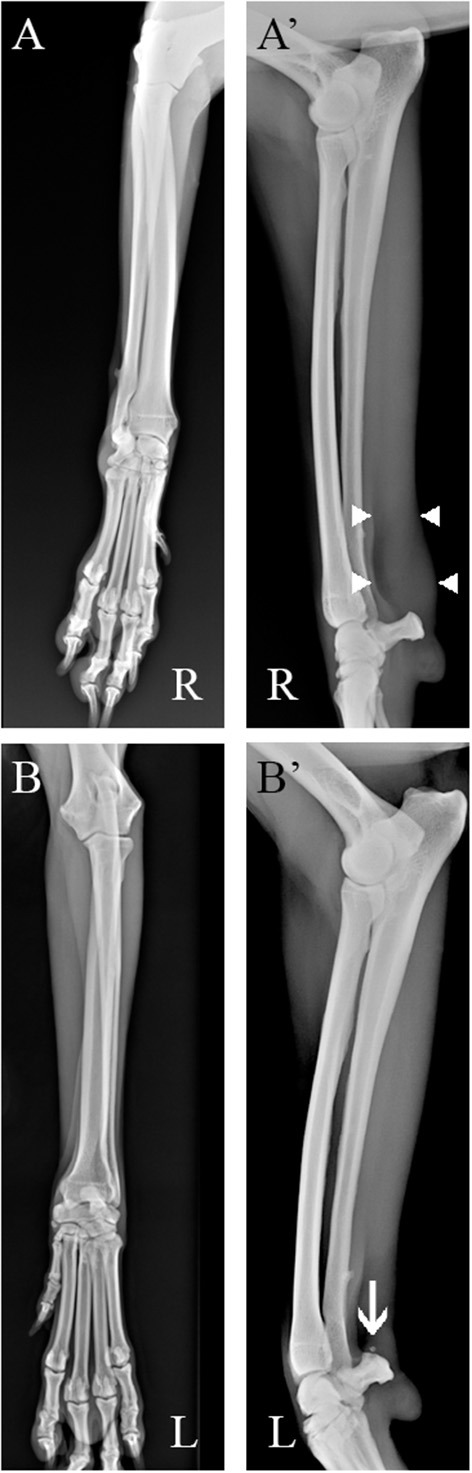
Radiographic assessment of the patient **(A)** Right side. Soft tissues thickening in the projection area of the FCU tendon, proximal to the accessory carpal bone [**(A′)**, between white arrowheads] are detectable in the mediolateral standard view **(B, B')** Left side. Entensiophites are detectable at the proximal pole of the accessory carpal bone (white arrow). No changes were detected on both craniocaudal standard views.

Radiographic examination of both carpal joints on the mediolateral and craniocaudal standard views was performed without the need for anesthesia and showed soft tissue thickening in the projection area of the right FCU tendon, just proximal to the accessory carpal bone. Enthesophytes were detected at the proximal pole of the left accessory carpal. No changes were detected bilaterally on the craniocaudal standard view (Figure 1).

### 2.3. Diagnostic imaging: Ultrasonographic evaluation

**Figure 2:**
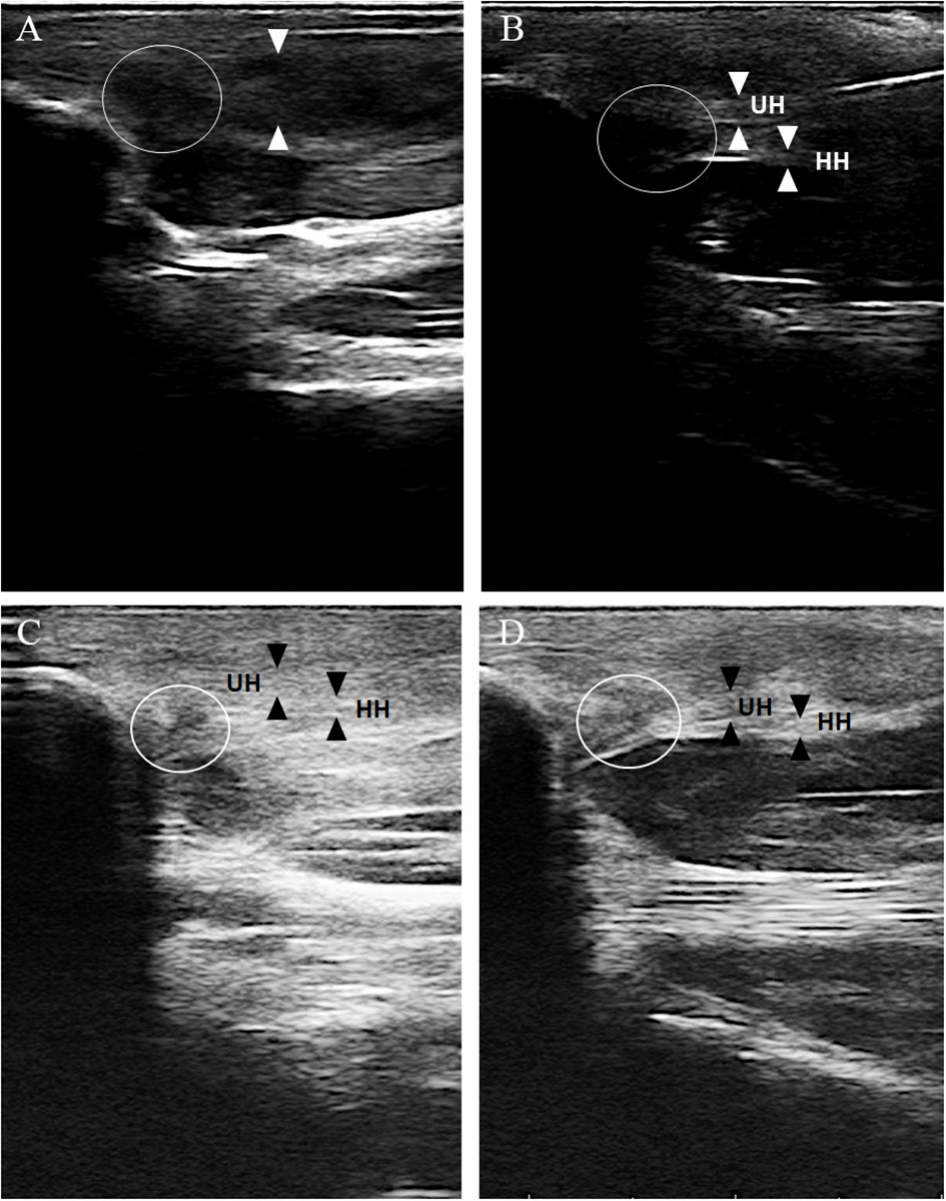
The ultrasonographic evolution of the healing process at different time points. **(A)** Ultrasonographic evaluation at T0, longitudinal scan. The ulnar and the humeral head of the right FCU tendon (between white arrowhead) can not be distinguished and shows ES 3 and FAS 3 (< 25% parallel fiber bundles) at its insertional portion on the accessory carpal bone. A hypoechoic area with complete loss of fiber alignment is detected (white circle). **(B)** Ultrasonographic evaluation at T1, longitudinal scan. The ulnar (UH) and the humeral (HH) head of the right FCU tendon show at its insertional portion on the accessory carpal bone an ES 2 and a FAS 2 with parallel fiber bundles between 25 and 49%. The hypoechoic area with complete loss of fiber alignment is still visible (circle) and consistent with T0. **(C)** Ultrasonographic evaluation at T2, longitudinal scan. The ulnar (UH) and the humeral (HH) head of the right FCU tendon show at its insertional portion on the accessory carpal bone an ES 1 and a FAS 2 with parallel fiber bundles between 25 and 49%. The hypoechoic area with complete loss of fiber alignment is still visible but smaller in diameter (circle). **(D)** Ultrasonographic evaluation at T3, longitudinal scan. The ulnar (UH) and the humeral (HH) of the right FCU tendon show at is insertional portion on the accessory carpal bone an ES 1 and a FAS 1 with parallel fiber bundles between 50 and 75%. The hypoechoic area with complete loss of fiber alignment is still visible and consistent with T2 (white circle).

Ultrasonographic evaluation of both carpal joints was performed using an 18 MHz linear transducer probe (MyLab SigmaVET, Esaote, Italy). For each assessment, a complete examination of the FCU tendon was performed using both longitudinal and transverse scans. The obtained images at each examination were evaluated and scored from 0 to 3 for two parameters, echogenicity score (ES) and fiber alignment score (FAS), modifying a previously published ultrasonographic assessment scale (20) (Table 2). The insertional portion of the right FCU tendon showed an ES of 3 compared to the contralateral one that had ES 1 without the presence of core lesions and an FAS of 3 with < 25% of parallel fiber bundles at the insertion on the accessory carpal bone. The contralateral group had FAS 1 with parallel fiber bundles at the insertion from 50 to 74%. Changes were detected in both the ulnar and humeral head of the tendon. Figure 2 shows the ultrasonographic appearance of the injured tendon at T0 and the evolution of the healing process during the PRP and PL administration program.

**Table 2:** Ultrasound assessment system (20).

**Parameter**	**Score system**	**Definition**
Echogenicity	0	Normal echogenicity
	1	Mildly hypoechoic
	2	Moderate hypoechoicity
	3	Severe hypoechoicity
Fiber alignment	0	≥75% parallel fiber bundles at the insertion site
	1	50–74% parallel fiber bundles at the insertion site
	2	25–49% parallel fiber bundles at the insertion site
	3	≤ 25% parallel fiber bundles at the insertion site

The clinical, radiographic, and ultrasonographic findings led to the presumptive diagnosis of FCU insertional tendinopathy. Because of the failure of conservative treatment, a regimen involving a PRP injection followed by two subsequent PL injections was suggested.

### 2.4. Treatment and follow-up

Blood samples (30 mL) were obtained from the jugular vein using an ACD-A 10% tube (BD Vacutainer^®^, BD, Italy). The treatment program consisted of three ultrasound-guided injections of autologous PRP prepared as previously described (21). Briefly, PRP was obtained following a double centrifugation tube method with first centrifugation at 2,800 rpm for 20 min and subsequently at 1,300 rpm for 15 min (TD4A-WS Desk Centrifuge, Drawell, China). All procedures were performed under sterile conditions. Three solutions of PRP (1 mL) were obtained at the end of the procedure. A blood count examination was performed on the first whole blood sample to assess the baseline platelet concentration of the dog, which was 165 K/μL. A subsequent blood count exam was made on the PRP sample having a platelet concentration of 1,029 K/μL assessing the achievement of a more than 5x platelet concentration for the final PRP preparation and a low concentration of white blood cells with 1,82 K/μL vs. 8,39 K/μL of the whole blood sample (BC-2800Vet, Mindray, China). Two of the three obtained PRP samples were frozen at −20°C and subsequently thawed for further administration of the PL (22).

On the day of the first injection (T0), an ultrasonographic examination was performed to assess the ultrasonographic appearance of the FCU tendons bilaterally. Before injection, no anesthesia was required, and the injection area, which was already clipped for ultrasonographic examination, was aseptically prepared. The treatment was inoculated in both heads of the right FCU tendon using a sterile 22G needle *via* ultrasonic guidance. Afterwards, only rest was prescribed, and short leash walks were allowed three times per day in the following 3 weeks.

The second and a third injection were performed at three (T1) and 6 weeks (T2), respectively, following the same protocol used for the first treatment and using the obtained PL (after freeze-thaw cycle) to continue the stimulation of the healing process.

## 3. Outcomes

The clinical and US outcome are summarized in Table 3.

**Table 3:** Clinical and US outcomes from T1 to T5.

**Time**	**Clinical outcome**	**US outcome**
T1	Decreased pain at palpation Decreased swelling of soft tissues Lameness score 2/4	ES 2 FAS 2
T2	No pain at palpation Mild swelling of soft tissues Lameness score 1/4	ES 1 FAS 1
T3	No pain at palpation No swelling of soft tissues Lameness score 0/4	ES 1 FAS 1
T4	No pain at palpation No swelling of soft tissues Lameness score 0/4	ES 1 FAS 1
T5	No pain at palpation No swelling of soft tissues Lameness score 0/4	ES 1 FAS 1

ES, Echogenicity score; FAS, Fiber alignment score; T1, week three; T2, week six; T3, week twelve; T4, week fifty-two, T5, week on-hundred and four; US, ultrasonographic imaging.

### 3.1. Clinical evaluation

Clinical assessments were performed every 3 weeks starting from the day of the first injection to the day of the last injection and every 12 weeks thereafter. In the present study, we report the clinical outcomes on the day of the first injection (T0), at three (T1), six (T2), twelve (T3), fifty-two (T4) and one-hundred-and-four (T5) weeks.

Before treatment, the dog presented with third-degree (3/4) right forelimb lameness, swelling of the palmar surface of the carpal joint, and pain on palpation of the affected region.

At T1, pain on palpation and swelling of the carpal region were slightly decreased; moreover, a reduction in the degree of lameness was observed (grade 2/4). The second injection was administered on the same day and it consisted of PL. At T2, the last PL injection was administered, and no pain on palpation was detected, with mild swelling of the carpal region. A grade 1/4 of lameness was recorded. At T3, 3 months after the first injection, no pain on palpation or swelling of soft tissues was detected, and grade 0/4 lameness was observed. T4 and T5 clinical examinations were consistent with T3 findings, with a complete restoration of forelimb function and return to sport. No reinjuries were reported.

### 3.2. Ultrasound evaluation

Ultrasound evaluation images are shown in Figure 2. At T0, before treatment, the US evaluation showed an ES 3 compared to the contralateral one that had ES 1 without detected core lesions and an FAS 3 with < 25% of parallel fiber bundles at the insertion on the accessory carpal bone. The contralateral group had FAS 1 with parallel fiber bundles at the insertion from 50 to 74%. Changes were detected in both the ulnar and humeral head of the tendon.

At T1, the US examination was mildly improved with ES 2 and FAS 2 compared to the contralateral one, which was still consistent with T0. Doppler ultrasound examination revealed the presence of a small amount of neovascularization. At T2, the US examination showed additional improvement with ES 1 and FAS 1 compared to the contralateral limb: T3, T4, and T5. US evaluation showed ES 1 and FAS 1 with the same ultrasonographic appearance for the FCU tendons of both forelimbs.

## 4. Discussion

FCU tendinopathy is poorly described in veterinary literature and rarely reported as a cause of forelimb lameness in pet dogs but is listed among the typical injuries of lure-coursing dogs (2). Its diagnosis is and will remain challenging as long as the disease continues to be considered underestimated and underrepresented. Working and sporting dogs are prone to FCU tendon injuries due to the repeated stresses they undergo during performance, leading to chronic tendon strain injuries. The etiology of FCU tendinopathy is poorly described in veterinary sports medicine, whereas in human medicine, it is thought to be related to repeated strain activity and overuse due to chronic repetitive movements (23). Regarding other pathologies involving tendinous structures, misdiagnosis or unsuccessful treatments can lead to a greater chance of re-injury, thus, becoming potentially career ending for canine athletes.

In the One Health scenario, the investigation of the treatment of flexor tendon diseases in dogs can be useful for the collection of preclinical data. The flexor tendon system of dogs has often been studied as an animal model for the investigation of the flexor tendon healing process, and the results have already been applied in human medicine (24). Moreover, the regenerative approach seems to be promising for tendon pathology management in both veterinary and human medicine, since PRP is currently the most exploited strategy in human clinical practice to provide a regenerative stimulus for tendon healing. In veterinary medicine, especially in canine athletes, the administration of PRP in combination with intratendinous injection of mesenchymal stem cells has demonstrated clinical benefits in other tendinopathies with minimal invasiveness (25).

Nevertheless, the lack of standardization of PRP administration modality represents an important aspect to evaluate in both human and veterinary approaches (26).

In the present case report, an ultrasound-guided peritendinous injection of autologous PRP followed by two further injections of PL was administered for the treatment of chronic right FCU tendinopathy in a lure-coursing dog. The patient presented with chronic lameness and ultrasonographic signs of FCU tendinopathy that developed during sporting activity and was unresponsive to previously administered conservative standard treatments such as rest, splint immobilization, and NSAIDs oral administration. The lack of response to the conservative approach and the risk to develop gastro-intestinal tract problems with a long-term treatment with NSAIDs were the two pressing elements of the management of the injury. Thus, the peritendinous injection of 1 mL of autologous PRP, followed by two further administrations of 1 mL per injection of PL, was the novel approach of choice. Different studies have shown the beneficial effects of PRP and PL on tendon healing in veterinary medicine (27–33). The positive action has to be ascertained to the concentrated growth factors and cytokines present in PRP/PL, which contribute to tenocytes recruitment and proliferation; moreover, they stimulate injured tenocytes to secrete angiogenic factors (34, 35).

The results were beneficial for the dog, as the applied therapeutic program resulted in the disappearance of lameness and ultrasonographic improvement of fiber alignment along with echogenicity of the injured tendon. Moreover, during the treatment and follow-up period, the patient did not suffer from any re-injury episodes; after T4, the dog returned to the same competition level before injury. The ability to return to sports could suggest restoration of the biomechanical properties of the tendon after the injections to an adequate level that allowed the dog to return to competition. This might be related to the supposed capability of low WBC PRP to support the production of collagen type I (COL1) fibers during the tendon healing process. COL1 is the major component of fibrillar collagen in tendons and is responsible for linear fiber alignment. On the contrary collagen type III, that is synthetized during primary tendon healing process, leads to the formation of disorganized scar tissue, which is more prone to re-injury in cases of excess load (36). The subsequent administration of PL at an interval of 3 weeks might have prolonged the activation of the healing process, leading to a physiological grade of fiber alignment and a good improvement in symptoms. Finally, repeated injections of autologous PRP and PL for the treatment of FCU tendinopathy in dogs did not provoke any adverse or immune reaction to the patient, supporting the safety of the treatment of choice.

The US observations of the process are another important aspect of the present case report because, to our knowledge, no studies have been published on US evaluation of the healing process of FCU tendinopathy. Musculoskeletal ultrasonography is an imaging technique that has been used and validated for the diagnosis of several tendinopathies in both humans and dogs. It provides a non-invasive diagnosis and allows for feasible and cost-effective consecutive examinations to assess the response to treatment; moreover, it is useful for evaluating the changes in size, shape, and echogenicity of the tendon, avoiding patient sedation (37, 38). Ultrasonographic evaluation of the healing process and prolonged US follow-up over time led to a controlled progression of the fiber alignment, which is an important element in the return-to-play decision-making process. Thus, as in human and veterinary medicine, high-frequency ultrasound imaging can be considered a useful tool for planning a return-to-play evaluation protocol for FCU injury treatment.

To our knowledge, no studies on the successful treatment of a lure-coursing dog's FCU tendinopathy by multiple ultrasound-guided PRP and PL administration have previously been published. The feasibility of this therapeutic approach could be considered satisfactory and safe in this case, as the dog returned to competition without any adverse treatment reactions. The ultrasonographic evaluation of the healing process played a pivotal role in a return-to-play oriented approach, and our results might be considered encouraging for the study of a more standardized application protocol of PRP administration for canine athletes, helping to collect preclinical data for human research.

## Data availability statement

The raw data supporting the conclusions of this article will be made available by the authors, without undue reservation.

## Ethics statement

Ethical review and approval was not required for the animal study because the owner of the dog signed a written consent. Written informed consent was obtained from the owners for the participation of their animals in this study.

## Author contributions

AF and ME followed the clinical case and performed the PRP and PL injections. MP and LM contributed to study design and supervised the study. ME, EC, and LM wrote the original draft of the manuscript. AF, ME, EC, LM, and MP revised and edited the manuscript. All authors read and approved the final manuscript.
